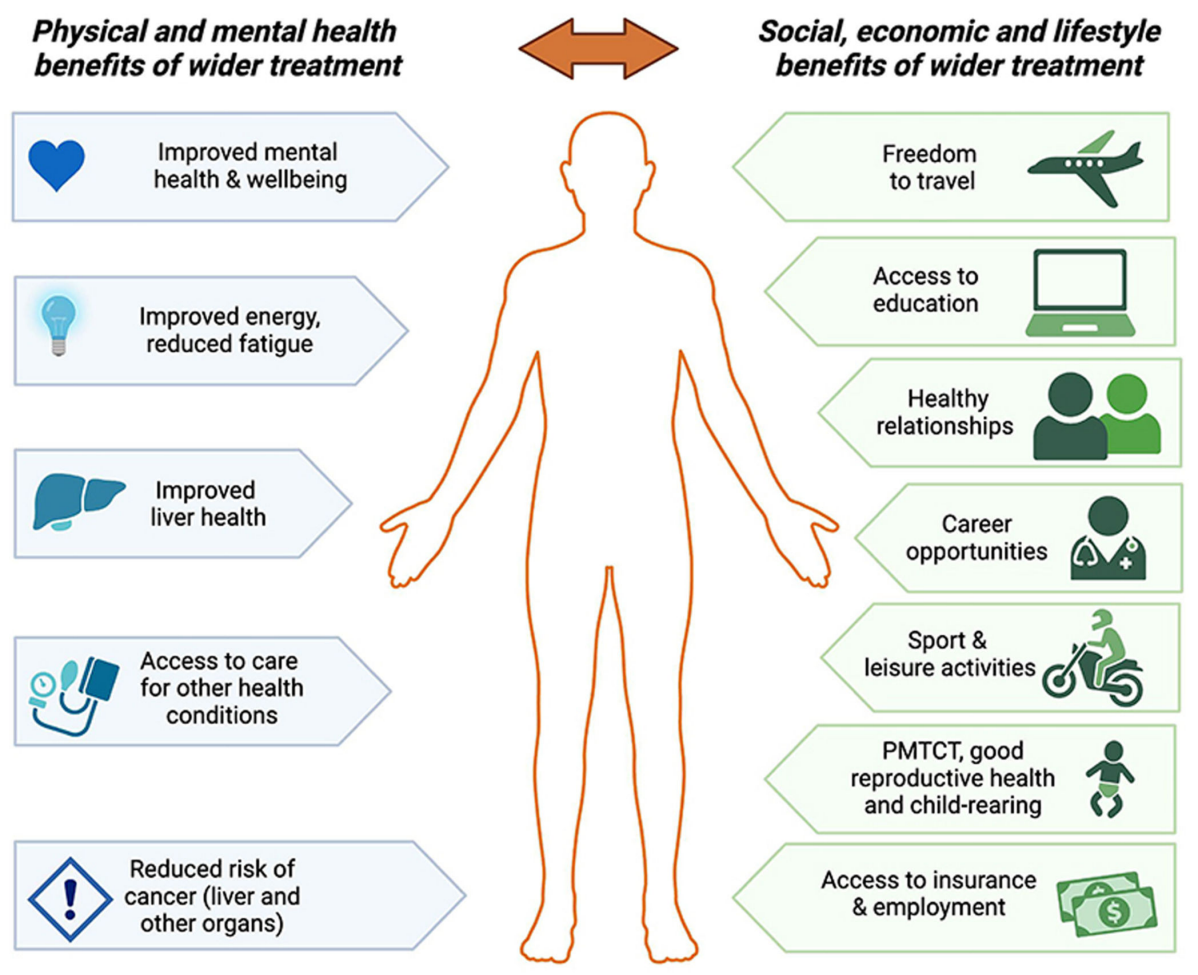# Patient and Public Health Perspectives to Inform Expansion of HBV Treatment Guidelines

**DOI:** 10.1016/S2468-1253(25)00052-4

**Published:** 2025-07-23

**Authors:** Chari Cohen, Thomas Tu, Philippa C. Matthews, Su Wang, Jessica Hicks, Manal El Sayed, John E. Tavis, for the International Coalition to Eliminate HBV (ICE-HBV)

**Affiliations:** 1 Hepatitis B Foundation, Doylestown, PA, USA; 2 Westmead Institute for Medical Research, University of Sydney School of Medicine and Health, NSW, Australia; 3 The Francis Crick Institute, London, UK; Department of Infectious Diseases, University College London Hospitals, London, UK; Division of Infection and Immunity, University College London, London, UK; 4 Center for Asian Health & Viral Hepatitis Programs, Cooperman Barnabas Medical Center, Livingston, NJ, USA; 5 World Hepatitis Alliance, London, UK; 6 Department of Paediatrics, Faculty of Medicine, Ain Shams University, Cairo, Egypt; 7 Department of Molecular Microbiology and Immunology, Saint Louis University School of Medicine, Saint Louis, MO USA; Saint Louis University Institute for Drug and Biotherapeutic Innovation, Saint Louis, MO, USA

## Abstract

Chronic hepatitis B (CHB) is associated with significant morbidity and mortality worldwide. People living with hepatitis B (PLWHB) face physical, emotional, social, and professional impacts reducing their quality of life. Treatment with nucleos(t)ide analog (NA) drugs reduces risk of cirrhosis and liver cancer and improves quality of life. However, few people globally are offered and can access affordable, long-term NA treatment. Moreover, limited progress has been made towards meeting hepatitis B elimination targets. The global landscape has been shifting towards expanding treatment criteria, but there has been little discussion about patient and community perspectives. We must view CHB treatment eligibility from a public health and patient-centered approach. Here, we discuss potential benefits and risks, implementation considerations, and public health questions and data needs surrounding expanding treatment eligibility for CHB. We conclude there is strong public health and community rationale for expanding treatment eligibility for PLWHB.

## Introduction

Chronic hepatitis B (CHB) is a liver disease caused by hepatitis B virus (HBV) infection, which significantly increases the risk of cirrhosis, hepatocellular carcinoma (HCC), and premature death.^1^ People living with CHB (PLWHB) also face physical, emotional, social, and professional impacts that lead to reduced quality of life.^2–6^ Physical symptoms commonly include fatigue, but chronic pain has been documented, and both limit participation in social activities and negatively impact job and familial capabilities.^6,7^ The fear and anxiety associated with disease progression and/or concern about transmission to others can lead to social isolation and depression.^2^ Stigma exacerbates withdrawal from society and discrimination limits education, career, travel, and economic opportunities and advancement.^7–13^

Treatment with currently approved first-line nucleos(t)ide analogs (NA) entecavir (ETV), tenofovir disoproxil fumarate (TDF) and tenofovir alafenamide (TAF) can prevent cirrhosis, and reduce the risk of liver cancer by up to 60%,^14^ and some studies have shown viral suppression with treatment to be associated with improved quality of life indicators.^15,16^ There has been a recent shift towards expanding hepatitis B treatment eligibility in CHB, seen in both the 2022 Chinese Medical Association (CMA) guidelines,^17^ which expand eligibility to people with any viremia and abnormal liver enzymes, and the 2024 World Health Organization (WHO) guidelines which expand eligibility to more than 50% of PLWHB (WHO recommends NA treatment for PLWHB age 12 and over with evidence of fibrosis or cirrhosis based on any of the following: APRI score >0·5; transient elastography value of >7·0 kPa; HBV DNA >2,000 IU/mL and ALT above upper limit of normal; coinfection with HIV, HDV or HCV, family history of liver cancer or cirrhosis, immune suppression; comorbidities such as diabetes or steatotic liver disease; or persistently abnormal alanine transferase (ALT) in the absence of access to HBV DNA assays).^18^ While we see this as progress, other professional societies have current guidelines that recommend treatment only for 12%-25% of PLWHB.^19–21^

The recent changes in the CMA and WHO guidelines are intended to address the concern that globally, we have not moved the needle on the hepatitis B cascade of care in decades. In 2022, among 254 million people with chronic hepatitis B virus infection, only 13% had been diagnosed and 2·6% (19·5% of those diagnosed) were receiving antiviral therapy.^22^ Additionally, there has been a striking increase in hepatitis B-related mortality, from 820,000 people in 2019 to 1·1 million people in 2022, making it the 3rd leading cause of infectious disease mortality (with only COVID-19 and tuberculosis causing more global deaths).^22–24^

The paradigm shift of treatment expansion involves expanding eligibility to initiate NA treatment earlier and for more people. This paper, commissioned by the International Coalition to Eliminate HBV (ICE-HBV), discusses the public health and patient-centered rationales for expanded NA treatment in PLWHB (Figure 1.). This includes the potential benefits and risks, implementation considerations, and public-health questions and data needs surrounding expansion of treatment. A companion paper also commissioned by ICE-HBV (Scientific and Medical Evidence Informing

Expansion of HBV Treatment Guidelines) highlights the scientific evidence for expanded treatment. In both papers, we have defined expanded treatment as follows: All people living with chronic hepatitis B with any detectable hepatitis B viral DNA in the blood should be considered eligible for treatment with currently available therapeutics, if any one of the following criteria is met: they request treatment, they have a family history of cirrhosis or HCC, they are older than 30 years of age, they have evidence of liver inflammation or liver damage, or they have co-morbidities or other risk factors that support treatment. This definition changes the paradigm from current guidelines, which require evidence of liver damage to be present before recommending treatment, and do not take patient preference into account when overt liver disease is not present. The definition we chose also negates the need for multiple longitudinal HBV DNA (or ALT) tests in determination of treatment eligibility. This definition is not intended to supersede professional medical society or organizational guidelines, rather is intended for use alongside clinical and laboratory assessment, is supported by scientific evidence and expert opinion, and reflects the voices and experiences of PLWHB.^25^

The conversation around expanding treatment eligibility for PLWHB has been evolving for the past few years, and there have been published reviews and commentaries discussing the clinical outcomes perspective, cost perspective, scientific perspective, and feasibility perspective.^26–28^ This paper reflects a new perspective – that of PLWHB. This perspective has been under-represented in the treatment expansion discussion. It is notable that virtually all HBV treatment and management guidelines still focus on clinical and lab-based indicators of chronic hepatitis B infection, without addressing quality of life impact, or accounting for concerns and preferences of PLWHB. It is critical that we adequately represent the perspectives of PLWHB and take strong action to improve the hepatitis B cascade of care and work towards global viral hepatitis elimination efforts from a public health and person-centered approach.

## A The rationale for expanding HBV treatment eligibility

### A1 Clinical health rationale

Expanded, more liberal consideration of treatment (beyond the scope of current professional society guidelines) could have a broad range of improvements on the health of PLWHB. Traditional outcomes of treatment are primarily related to reducing morbidity and mortality from liver related complications such as cirrhosis and HCC. However, it can take years of NA treatment to deliver on the impact of HCC.^29^ As pointed out in the companion paper by Kennedy *et al*., there is ample evidence that people who fall outside treatment guidelines still develop HCC.^30–35^ For example, a recent study found that among a cohort of 3,264 treatment-naïve PLWHB, 4·4% developed HCC over the 4·6 year follow up period, and a large proportion of them fell outside the treatment guidelines from EASL (34%), APASL (64%) and AASLD (46%).^33^ Additionally, long-term HBV replication and DNA integration drive pathogenesis, which can be suppressed with NA treatment.^29^ HCC-associated DNA damage occurs early in infection and requires timely intervention to be prevented.^36^ While certain HCC risk factors such as age, gender, and viral genotype are not modifiable, HBV replication and HBV DNA levels can be reduced by NA treatment. Therefore, expanding treatment guidelines towards earlier treatment initiation based solely on the outcome of preventing oncogenesis is justifiable.

There are also emerging data on non-hepatic malignancies related to HBV including gastrointestinal (stomach, colon, pancreatic), neuroendocrine and gynecologic cancers, and lymphoma).^37–39^ It remains unknown whether these also may be mitigated by NA therapy, though one cohort study in Korea found that complete viral suppression with long-term NA treatment reduced incidence in some PLWHB.^37^

### A2 Quality of life rationale

In addition to reducing the risk of cirrhosis and HCC, improving quality of life dimensions should be considered important reasons for initiating therapy. Quality of life has been increasingly studied in hepatitis B and results show that PLWHB have reduced quality of life encompassing social, emotional, and physical wellbeing.^2–6^ This includes impacts related to fear of disease progression, anxiety about a possible shortened life, and concerns about transmitting to others.^5–7,11,12,40^ Pain and fatigue are also commonly reported by PLWHB in surveys and interview data, yet HBV is largely considered asymptomatic for noncirrhotic individuals, and physical symptoms are not currently an indication for treatment.^6,7,12^ As laid out by Tu *et al*. in a classification of primary, secondary and tertiary quality of life impacts, there is a complex interplay with internal views of self from having hepatitis B and these impact one’s interactions with immediate community and then interactions with broader society.^11^

Many PLWHB have normal liver enzymes and are otherwise considered asymptomatic by clinicians. However, an individual’s experience of living with hepatitis B can lead to a great deal of self-stigma, shame, guilt and worry that is not shared with clinicians.^6,9,13,41^ The fear of liver cancer, cirrhosis, and transmission can impose a heavy mental health burden.^6,7^ This aspect of the impact of hepatitis B is not consistently assessed in clinical settings, but surveys of PLWHB show that this “worry index” is substantial.^6^

The risk of transmitting HBV can keep people from intimate relationships and many individual stories document this kind of devastating impact (rejection by partners, divorces, abandonment, forced abortions, decisions not to have children).^7^ Because of self and external stigma, discrimination and fear of transmission, PLWHB can withdraw from social interactions and relationships.^13^

Finally, hepatitis B may impact an individual’s interactions with wider society, which can influence their career, livelihood, wellbeing and relationships with the community.^42,43^ Many stories document the negative impact of hepatitis B on education, jobs and social and personal relationships.^7,27,28^ In some situations, plans for immigration have to be abandoned, influencing opportunities for employment, career development, study and personal autonomy.^42,43^ Documented cases of job discrimination, particularly relating to healthcare workers and the military, have been documented with the U.S. Department of Justice, and collected by the Hepatitis B Foundation discrimination registry.^42–44^

NA therapy can play a positive role in ameliorating quality of life impact for PLWHB. Patient Reported Outcomes (PROs) in surveys of people who are virally suppressed due to receiving NA therapy show improved quality of life, including improved physical symptoms and emotional functioning, and less fatigue and worry.^15,16^ This evidence, alongside data showing that PLWHB exhibits reduced quality of life impact in the absence of liver damage or liver disease,^40^ provide a strong rationale for expanding treatment eligibility. Additionally, PLWHB on NA medication typically have more regular medical follow up visits due to the need for blood tests and refills. This increased engagement and clinical oversight could also mean better overall health due to regular care, but this needs to be better studied.

### A3 Reducing transmission anxiety rationale

In addition to reducing individual risk of complications, expanding treatment guidelines is also likely to limit transmission of hepatitis B, along with related anxiety. Indeed, currently implemented guidelines already advocate NA use to prevent transmission in limited circumstances where transmission risk is considered high, including for preventing mother-to-child (perinatal) transmission, and health care workers conducting exposure prone procedures.^17–21^ However, the guidelines do not recommend treatment based on potential risk of transmission in other contexts (e.g., tattooing, unprotected sex, intravenous drug use, etc.). Infection prevention recommendations have focused primarily on perinatal prevention and childhood vaccination, along with recommendations for promoting safe injection practices in healthcare, and preventive behaviors such as barrier use during sexual contact and sterile equipment for injection drug use or tattooing.^45–47^ While vaccination and behavioral-based interventions are recommended, their implementation varies widely by region^22^, and they may not cover all instances or activities (e.g., contact sports). This can lead to a heavy and often impossible-to-implement burden of responsibility on PLWHB to ensure the vaccination of those around them, or refrain from participating in social situations if they are unsure about transmission risks. Expanding treatment eligibility would provide an additional choice for PLWHB to increase assurance of limiting transmission risk by using multiple interventions, increase the number of interventions that can fit into different lifestyles and contexts, and reduce self-abstinence of social interactions out of anxiety over transmission risk.

Ensuring vaccination of those potentially exposed requires difficult conversations and possibly disclosure of one’s own hepatitis B status, exposing PLWHB to stigma and discrimination. Moreover, there may be several factors leading to vulnerability to infection even if this conversation is carried out, including vaccine hesitancy or unwillingness to be vaccinated, mis-remembering vaccination status, non-response to the vaccination, etc. Finally, ensuring a full vaccination course and a seroprotective antibody response level prior to potential exposure is impractical or impossible in many scenarios (such as waiting a full 6 months before unprotected intimate contact). In summary, current approaches do not bring to bear the full range of precautions to prevent transmission and the existing measures to reduce transmission are not infallible.

Reduction of transmission is of particular concern during adolescence and young adulthood. Most adolescents and young adults living with hepatitis B have been untreated despite high viral load, as they fall outside treatment criteria outlined by most national society guidelines to date.^48^ This puts them at greater risk of transmitting the virus during coming-of-age eras that include social and sexual exploration^49^ and is a concern for both sexual and perinatal transmission. Every year, an estimated 12 million adolescents between the ages of 15-19 give birth, primarily in resource limited settings.^50^ There is a heavy burden for young people with hepatitis B, who deal with fears of transmitting the virus, along with the tribulations of navigating disclosure and facing discrimination and isolation. WHO guidelines have expanded treatment down to those age 12 years, which is a positive advance in meeting the needs of this population.^18^ However, it is worthwhile re-framing discussion of treatment for young people to include the goal of reducing viral loads before initiation of higher-risk behaviors associated with adolescence.

No amount of information and caution can cover every possible exposure event, and the affected community should not be expected to abstain from all activities that could potentially lead to transmission. There may be activities that depend on a large range of factors as to its risk of transmission but for which data are very poor (e.g., contact sports, minor injuries, wet shaving using a razor). Treatment access, for people seeking it, would provide an extra layer of prevention in horizontal transmission and allow the affected community to participate in life with less fear.

We can learn from the HIV community and the tremendous progress made towards reducing both transmission and stigma. Some countries have been able to significantly reduce new HIV infections by implementing a combination of primary prevention including pre-exposure prophylaxis PrEP and expanding access to antiviral treatment.^51,52^ We can also learn from the power of the “Undetectable = Untransmittable” (U=U) paradigm, which was integral to the reduction of stigma and discrimination for HIV.^53,54^ This messaging states that when a person living with HIV maintains an undetectable viral load due to antiviral suppression, they cannot transmit the virus via sexual contact.^53^ The hepatitis B field could similarly adopt a properly qualified message similar to U=U. Current guidelines for the management of infected healthcare providers accept that there is a negligible risk of transmitting HBV due to exposure to biological fluids during an invasive surgical procedure if the provider has an undetectable viral load.^54^ Despite scant studies showing this, it is highly likely that U=U largely holds for HBV transmission during sexual contact. Evidence for this will be difficult to collect due to the ethical obligation to offer vaccination to partners of PLWHB.

Finally, the reduction in transmission anxiety by antiviral treatment is seen as a benefit for other conditions. Reductions in HIV transmission anxiety have been documented in people taking oral PrEP, and it is likely similar reductions would be seen for those affected by HBV.^55,56^ Qualitative and quantitative data collection have found that decreasing anxiety about transmitting to others is considered a primary benefit of taking NA treatment among PLWHB.^7^ Moreover, the attitudes of physicians prescribing PrEP for HIV are in line with this view, supporting prescription of PrEP to reduce anxiety (if there were no medical contraindications) independent of the actual transmission risk.^57^ Similar practices should be considered for PLWHB.

Despite current prevention strategies, there were 1·2 million new global HBV infections in 2022, and despite a lack of surveillance to assess the proportion of these that were not the result of perinatal transmission, it is clear that horizontal transmission plays a role.^22^ In intermediate and low endemicity regions, according to WHO, ”a substantial disease burden may result from acute and chronic infection acquired by older children, adolescents and adults.”^18(p. 10)^ Expanding hepatitis B treatment in combination with adopting properly qualified U=U messaging and increased use of the hepatitis B vaccine can significantly enhance efforts to reduce transmission rates, and has the potential to reduce fear and stigma, and prevent discrimination in contexts including personal relationships, participation in sports, migration, insurance, and employment.

## B Implementation considerations

Changing treatment guidelines alone will not improve the health of PLWHB, but should be part of a wider program to expand access to care. Expanding treatment eligibility increases the choices that PLWHB have to manage their condition, but is only a single component of an effective public health response.

Expanded and more inclusive treatment will require new models of care delivery with decentralization of services, and task-shifting to frontline providers in order to improve efficient, consistent, equitable delivery of diagnosis, treatment and linkage to care.^22^ Different models of care delivery may be of particular value for reaching vulnerable and marginalized populations and those at highest risk of complications.^22^ The involvement of primary care and community-based services in non-clinical centers provides opportunities to offer culturally sensitive education and advice, and to provide diagnosis and offer treatment to wider population groups. Providing hepatitis B care and treatment as part of other services removes silos between disease areas and allows for integrating hepatitis B as part of a “syndemic” of overlapping challenges, and allows for more holistic care of health and wellbeing.^58^ For example, hepatitis B screening is already recommended by WHO as part of antenatal care, and 75% of reporting countries report routine hepatitis B testing in antenatal settings.^22^ This is therefore an important entry point to delivery of maternal and child interventions in the peri-partum period and beyond. Hepatitis B care during pregnancy can go beyond the mother and infant and also extend to family members who may need testing and vaccination and thus widen the reach of HBV services. Additionally, a holistic approach promotes integrating hepatitis B services into care for other chronic conditions such as hypertension and diabetes, and into other settings, such as mental health, substance abuse treatment, harm reduction, sexual health, and migrant health. In parallel, scale-up of hepatitis B peer navigators and supporters can improve continuity of care and empower individuals to become more involved in taking autonomous treatment decisions.^59^

There is also a need for adolescent-focused services, and there are models of integrated care delivery for adolescents living with other long-term health conditions (perhaps best exemplified by HIV management programmes, but also including other chronic diseases) that could be adapted.^60^ Providing hepatitis B education, care and treatment into a decentralized adolescent care delivery system, especially in low- and-middle-income countries, can help ensure early and sustainable NA treatment. This can have an enduring positive impact for adolescents and the people around them.

The new WHO guidelines for the first time include adolescents over 12 as part of the same treatment recommendation as adults.^18^ They also recognize that HBV treatment can be prescribed in the form of fixed-dose combinations of agents (e.g. tenofovir combined with lamivudine). These formulations, while not available for HBV in pediatric formulations, have become widely available through HIV programs and thus offer improved access and affordability compared to tenofovir monotherapy, which is expensive or inaccessible in some settings.^18,22^ Roll-out of wider diagnosis and more permissive hepatitis B treatment is supported by the development, validation and implementation of new tools for more efficient, community based, approaches to diagnosis, assessment and surveillance. For example, diagnostic tools include point of care testing for HBV multiplexed with HIV, and syphilis, with evidence that combined diagnosis is a cost-effective approach to the global “triple elimination” agenda.^61^ Molecular tests to quantify HBV DNA are also available to provide portable, near-patient assessment, which allow quick treatment decisions and linkage to care.^62^ This approach may be of particular benefit in settings where current antiviral therapy for hepatitis B is cheaper and more accessible than the tests needed to gauge eligibility.^18^

Elimination goals and the agenda for wider treatment may also prompt increased interest in novel surveillance tools for hepatitis B, including novel biomarkers of risk for long-term liver complications including HCC.^63^ These strategies are currently out of reach for most populations due to access barriers and cost, and if they are to inform routine practice, tools must be made widely accessible and affordable.

Having more individuals engage in existing NA therapy expands the population who are in a position to receive the new therapies for hepatitis B under development that are aiming at functional hepatitis B cure because a common eligibility criteria for these new therapies is based on HBV DNA suppression while on NA therapy. Current hepatitis B therapy does not exist in long acting or injectable formulation, but development of such agents could be of benefit in many populations, in particular to support adherence. Thus, expanded use of existing therapy “with support for parallel monitoring and follow-up” readies individuals, populations and clinical services for the roll-out of new therapies, and could further incentivize investment from pharmaceutical companies, funders, and the clinical research community.

Expanding treatment eligibility globally can play a role in improving future access to screening, sustainable care, and treatment by creating demand, which should drive the market towards reduced diagnostic and NA costs, and drive governments and health care systems to implement policies and leverage integrated care delivery to improve access. Therefore, there is a practical, service-level beneficial impact of wider treatment, including enhancements in clinical service provision, improvement in access to robust screening and risk assessment, opportunities to participate in clinical trials, and development of novel treatment strategies. These advances can improve quality of life, promote engagement in long-term care and treatment, and support the delivery of equitable, cost-effective healthcare.

## C Risks of implementing expanded treatment

The benefits of expanded treatment need to be weighed against any risks, as is addressed in the companion paper by Kennedy *et al*. Current therapies for hepatitis B require lifelong treatment, and adherence to that treatment is important if people are to achieve the intended long-term clinical benefits. Incomplete adherence (compared to high adherence) for NA therapy among PLWHB has been poorly studied, but can lead to acute and negative health outcomes, including mortality.^48^ Expanding treatment can potentially lead to clinically significant hepatic flares for some who are unable to adhere to treatment, though there is wide heterogeneity related to the size of this risk among published studies. A recent systematic review and meta-analysis suggested limitations with much of the published literature and found that after pooling and carefully defining outcomes data, only 1.2% of patients in 15 studies developed hepatic flares or decompensation after stopping NAs.^64^ Therefore, we need to consider how these potential risks compare with the current oncogenic risks and substantial quality of life impact of not starting treatment at all among the large population of PLWHB who do not meet current treatment eligibility guidelines.

Being under 45 years of age and treatment naïve (compared to those re-starting treatment) is associated with lower adherence.^65,66^ As expanding treatment will likely impact younger people, the issue of adherence must be addressed if expanded treatment is to be implemented. Key to this is understanding the underlying factors that influence whether or not a person adheres to the prescribed treatment which can include anxiety about side effects, low motivation due to a perceived lack of efficacy or need, poor health literacy, out-of-pocket costs, stigmatization and cost barriers.^67,68^ These can be addressed through improving hepatitis B knowledge and communication skills of medical professionals, improving patient education, and ensuring that access to repeat prescriptions and ongoing monitoring is delivered in a way which is convenient, accessible and affordable for the patient.^69,70^

While adherence must be addressed if we are to expand treatment eligibility, we need to reconsider the evidence and the weight currently placed on adherence as a treatment barrier. Though there is little rigorous data assessing adherence to NA therapy among PLWHB, adherence concerns related to serious viral flares have been widely discussed as a barrier to expanding treatment eligibility. There are other chronic conditions for which long-term treatment is recommended (HIV, hypertension, diabetes), for which adherence is also a concern, where lack of adherence can lead to sometimes life-threatening disease outcomes. Yet these concerns have not limited treatment recommendations to the majority of those affected populations. Instead of these concerns resulting in limiting treatment recommendations, clinical recommendations provide guidance for managing adherence, with a focus on interventions that provide patients with support to improve access, uptake and adherence. For example, the international hypertension practice guidelines cite nonadherence in 10-80% of people diagnosed with hypertension, yet treatment is recommended along with lifestyle modification even for low-risk individuals, and recommendations include provider and patient-focused strategies to improve adherence.^71^ Why is adherence such a strong concern for initiating hepatitis B treatment when the risk of symptomatic flares are low,^64^ most flares are self-resolving, and close monitoring with timely re-initiation of treatment is effective for most people in preventing or reversing hepatic decompensation resulting from treatment cessation? We contend that we should be emphasizing how to maximize hepatitis B treatment access and adherence, and that broadening the conversation to manage and treat hepatitis B as an oncogenic process and an infectious agent could help overcome some of these long-held adherence-related beliefs that continue to stand in the way of expanding treatment.

A further risk to expanded treatment is increased exposure to potential side effects. While NAs are generally well tolerated, there can be side effects to TDF including bone and renal toxicity.^72^ There are few studies on the side effects of NA treatment for hepatitis B infection over the long term, however the same or similar NAs are used in HIV treatment which is also lifelong with people starting on treatment as soon as they are diagnosed. Based on robust safety data, and weighing risks with accessibility and efficacy, TDF is still recommended as part of combination therapy for most people with HIV.^73^ These data can be largely extrapolated from the HIV field as per clinical practice guidelines;^73^ a similar approach can be taken to safety data during pregnancy and breastfeeding.^74^ Additionally, as access to TAF for HBV treatment grows globally, both bone density loss and renal toxicity should be somewhat mitigated, as TAF has a better safety profile related to these side effects.^73,75^

Expanded treatment also has cost implications for PLWHB and the health system. The individual cost of treatment can impact both on decisions to start treatment and adherence to treatment, especially in the absence of symptoms. Analyses have been done showing that treating all can be cost effective and if yearly drug costs are $750 USD or less, it can actually be cost saving.^76^ More data are needed on the cost-effectiveness of an expanded treatment approach in different settings and countries.^76^ It is important to note that the most common formulations of TDF and ETV are generic off-patent. Although TDF can be procured for the global benchmark price of US $2·40 per month, currently few countries are paying at or below this price and end costs to individuals are often much higher.^18^ Countries should be encouraged to push for access at these prices, which have the potential to improve the cost-effectiveness of expanded treatment.

There is a theoretical potential that expanded treatment eligibility could fuel community pressure for PLWHB to be treated regardless of individual choice, thereby validating stigma and discrimination and embedding inequality. Moreover, PLWHB who do not have access to or choose not to take treatment could be discriminated against. These potential risks do highlight the necessity to advocate for education about transmission risks, broadening access to care and treatment, legal discrimination protection, implementation of universal precautions, etc. in addition to treatment eligibility expansion. However, it is widely accepted that the U=U campaign for HIV has led to a reduction (not increase) in stigma and discrimination and has been an important advocacy tool to increase access to treatment.^77^ Thus, the risk of misinterpretation should not necessarily be the cause for withholding treatment expansion to PLWHB, but should be mitigated with parallel public health actions.

In summary, we believe that access to treatment should not be limited as the sector waits decades for unimpeachable evidence of improved clinical outcomes and treatment adherence (which may not be realistic given the lack of funding for hepatitis B research and the ethical issues associated with multi-decade controlled studies). There is growing data showing the benefits of treatment on improving lives of PLWHB and the potential risks are uncommon. There are guardrails for addressing these including monitoring, changing the NA prescribed, patient choice, and determination of likely adherence before prescribing antiviral therapy. Implementation of expanded treatment needs to include provision of appropriate education and psychosocial support, and access to affordable treatment to maintain optimum adherence. Bolstering efforts of education, access to clinical monitoring, peer support, and simplified service delivery will be enablers for retaining people on NAs.

## D Public health-focused scientific questions and data needs

There is a growing body of evidence related to community and provider preference, implementation and adherence, cost and cost-effectiveness, and the role of transmission prevention, though more data are needed. Table 1 outlines important areas for focused research. Implementing studies to fill knowledge gaps will necessitate extensive resources and time. Looking at the overall lack of investment and prioritization for funding hepatitis B research, it is questionable whether funding will be made available to answer all or even most of the scientific and public health questions.^78^

This raises the question of what strength of evidence is necessary to recommend wider treatment, or to create more flexibility in treatment guidelines, that would enable providers to initiate earlier treatment, based on the needs and perspectives of PLWHB. As some of these questions can take many years to decades to answer definitively (and the ideal studies may be ethically impermissible), we question whether we have the luxury to wait for the strongest level of scientific evidence – or indeed, whether we will ever have such evidence. For example, it could take decades and millions of dollars to generate robust data assessing efficacy of current NAs in reducing cirrhosis and HCC risk among people who do not meet current treatment guidelines. Barring a large, long-term randomized controlled trial (RCT), what data could be leveraged in the near future? Perhaps the newly expanded Chinese guidelines offering treatment more broadly will generate data, but that in itself could take many years. Similarly, will we ever have enough evidence for the scientific and clinical community to be comfortable making treatment decisions based upon a “treatment as prevention” paradigm, and adopting an HBV-specific U=U message? Therefore, we ask what treatment recommendations can we reasonably make with data that are currently available, or based upon feasible, affordable studies that can be completed in a timely interval?

## Conclusion

This paper, and its companion manuscript, make the case that there is both public health urgency and scientific evidence that strongly support expanding hepatitis B treatment (Figure 1). While it is important to initiate studies that can advance the evidence base, we must also take a pragmatic view of real-world experience, feedback from communities and people with lived experience, and data from parallel fields. We need to consider the scientific evidence and public health/clinical data we already have, weighed against the critical need, to make informed and reasonable decisions that will help save and improve lives in a timely manner with the effective tools we currently have.

## Figures and Tables

**Figure 1:**